# Early Age of Onset Is an Independent Predictor for a Worse Response to Neoadjuvant Therapies in Sporadic Rectal Cancer Patients

**DOI:** 10.3390/cancers15143750

**Published:** 2023-07-24

**Authors:** Caterina Foppa, Annalisa Maroli, Antonio Luberto, Carlotta La Raja, Paola Spaggiari, Cristiana Bonifacio, Stefano De Zanet, Marco Montorsi, Salvatore Piscuoglio, Luigi Maria Terracciano, Armando Santoro, Antonino Spinelli

**Affiliations:** 1 Department of Biomedical Sciences, Humanitas University, Via Rita Levi Montalcini 4, 20090 Milan, Italy; caterina.foppa@hunimed.eu (C.F.); antonio.luberto@humanitas.it (A.L.); carlotta.laraja@humanitas.it (C.L.R.); marco.montorsi@hunimed.eu (M.M.); luigi.terracciano@hunimed.eu (L.M.T.); armando.santoro@hunimed.eu (A.S.); 2 Division of Colon and Rectal Surgery, IRCCS Humanitas Research Hospital, Via Manzoni 56, 20089 Milan, Italy; annalisa.maroli@humanitas.it (A.M.); stefano.dezanet@humanitas.it (S.D.Z.); 3 Division of Pathology, IRCCS Humanitas Research Hospital, Via Manzoni 56, 20089 Milan, Italy; paola.spaggiari@humanitas.it; 4 Division of Diagnostic Radiology, IRCCS Humanitas Research Hospital, Via Manzoni 56, 20089 Milan, Italy; cristiana.bonifacio@humanitas.it; 5 Division of General and Digestive Surgery, IRCCS Humanitas Research Hospital, Via Manzoni 56, 20089 Milan, Italy; 6 Institute of Medical Genetics and Pathology, University Hospital Basel, University of Basel, 4001 Basel, Switzerland; salvatore.piscuoglio@usb.ch; 7 Department of Biomedicine, University Hospital Basel, University of Basel, 4001 Basel, Switzerland; 8 Division of Medical Oncology and Hematology, IRCCS Humanitas Research Hospital, Via Manzoni 56, 20089 Milan, Italy

**Keywords:** early-onset, rectal cancer, late-onset, neoadjuvant therapies, age of onset, tumor regression grade

## Abstract

**Simple Summary:**

Early-onset rectal cancer (EORC) patients are more likely to undergo neoadjuvant therapies due to the advanced stage of cancer at diagnosis. The response to therapies in this group of patients is still unknown. The aim of this study was to explore the effect of age of onset on the pathological response to neoadjuvant therapies in locally advanced RC patients. A higher rate of incomplete response was reported in EORC patients and early age of onset presented as a risk factor for a worse response in a multivariable analysis. The results of this study call for a different multimodal strategy in EORC patients.

**Abstract:**

The incidence of rectal cancer (RC) is increasing in the population aged ≤ 49 (early-onset RC-EORC). EORC patients are more likely to present with locally advanced disease at diagnosis than late-onset RC (LORC; aged ≥ 50) patients. As a consequence, more EORC patients undergo neoadjuvant therapies. The response to treatment in EORC patients is still unknown. This study aims to explore the effect of age of onset on the pathological response to neoadjuvant therapies in sporadic locally advanced RC (LARC) patients. Based on an institutional prospectively maintained database, LARC patients undergoing neoadjuvant therapies and radical surgery between January 2010 and December 2022 were allocated to the EORC and LORC groups. The primary endpoint was the rate of incomplete response (Dworak 0–2). A total of 326 LORC and 79 EORC patients were included. Pre-neoadjuvant tumor features were comparable. A significantly higher rate of incomplete response was observed in EORC patients (49% vs. 35%; *p* = 0.028). From multivariable analysis, early age of onset, smoking and extramural invasion presented as independent risk factors for a worse response. This study demonstrates that an early age of onset is related to a worse response and calls for different multimodal strategies in this group of patients.

## 1. Introduction

Early-onset colorectal cancer (EOCRC; age at diagnosis 49 or younger) has progressively risen worldwide (approximately 2% per year since 1994) [1,2,3,4], representing—over the past 30 years—the main cause for cancer death for men aged < 50. The increased incidence has been mainly observed in sporadic and left-sided (distal colon and rectum) cancers [5]. Early-onset rectal cancer (EORC) patients are usually diagnosed with a locally advanced disease [6,7,8,9,10]. As a consequence, EORC patients are more likely to undergo neoadjuvant therapies than older patients (late-onset rectal cancer, LORC; age at diagnosis 50 or older). Current guidelines on the management of RC are based on the evidence given by trials including mainly LORC patients [11] and do not differentiate the treatment according to age. The different biologic and molecular profiles between EORC and LORC patients [12] may have an impact on the response to preoperative regimens [13]. In fact, some authors [12,13] have suggested that the higher rate of signet ring cells, poorly differentiated tumors, mucinous histology and other features indicating a more “aggressive” nature seen in patients with EORC may explain the more aggressive tumor behavior in young patients. A national study analyzing 43,106 stage II and III RC patients treated according to the National Comprehensive Cancer Network guidelines did not report a survival benefit in young patients, suggesting a potential worse response to treatment [13]. The evaluation of treatment response and the identification of molecular features associated with response or resistance to neoadjuvant treatments in EORC patients is of utmost importance to improve the therapies in young patients with the final aim of improving survival. In fact, a study [14] reported that patients achieving a >95% response to preoperative multimodal treatments have an improved long-term oncologic outcome. Our group reported a worse pathologic response to neoadjuvant therapies in EOCRC patients [15]. To better explore this finding, we decided to compare the pathologic tumor response to neoadjuvant therapies in sporadic EORC and LORC patients, and to explore the factors associated with a worse response.

## 2. Materials and Methods

### 2.1. Study Design and Patients’ Selection

This is a single-center observational ambidirectional parallel-cohort study, conducted in a tertiary referral center. The study was approved by the local ethical committee. Consecutive patients diagnosed with rectal adenocarcinoma who underwent neoadjuvant therapies and curative rectal resection between January 2010 and December 2022 were included. Neoadjuvant therapies included standard chemoradiotherapy (CRT −45–50.4 Gy in 25–28 fractions concurrently with 5-fluorouracil or capecitabine) [16], short-course radiotherapy (SCRT −25 Gy in 5 fractions without CT) [17], total neoadjuvant therapy, TNT—standard CRT + induction or consolidation systemic chemotherapy with XELOX or FOLFOX or FOLFOXIRI regimens) [18]. Exclusion criteria were patients aged < 18, patients with metastatic RC (stage IV), any histological diagnosis different from primary rectal adenocarcinoma, patients undergoing local excision, patients enrolled in the watch-and-wait protocol after neoadjuvant treatment, inflammatory bowel diseases, genetic syndromes and a threshold of 5% of missing data. During the study period, five EORC patients have been enrolled in the watch-and-wait protocol, and three of these underwent surgery for regrowth while two are still disease-free. Eight LORC patients have been enrolled in the watch-and-wait protocol, and three of these underwent surgery for regrowth while five are still disease-free. To homogenize the results, patients enrolled in the watch-and-wait protocol and operated for a regrowth during the follow-up were excluded from the analysis. Patients were sorted in the study cohorts according to their age at the time of diagnosis: Early-Onset (≤49 years -EORC) and Late-Onset (≥50 years -LORC).

### 2.2. Endpoints and Variables

The primary endpoint was to compare the rate of incomplete pathological tumor response after neoadjuvant therapies in the EORC and LORC groups. Tumor response was assessed both on the primary tumor and on lymph nodes. The primary tumor was evaluated according to the Dworak tumor regression grade (TRG) [19]: a five-tier grading system ranging from 0 (no response) to 4 (complete response). A Dworak value of 0–2 corresponds to a complete lack of response or partial response and was therefore defined as “incomplete regression”; a Dworak value of 3–4 corresponds to a near complete or partially complete response and was therefore defined as “major regression” [19]. Additionally, the pathologic response on lymph nodes was evaluated and the two results were combined. A pathological complete response (Dworak 4) was defined as the absence of viable tumor cells in either the primary tumor site and the resected lymph nodes (ypT0N0). The secondary endpoint was to explore the effect of clinical and tumoral characteristics on the risk of achieving an incomplete tumor regression after neoadjuvant therapies.

Locally advanced RC was defined according to staging magnetic resonance imaging (MRI) as any T3/T4 cancers with negative local lymph nodes or any T with positive local lymph nodes. All cases were discussed at the multidisciplinary tumor board as per protocol of our institution.

Patients with at least a first/second degree relative for CRC—not fulfilling the Amsterdam or Bethesda criterion for hereditary non-polyposis colon cancer (HNPCC) syndrome or the clinical criterion for familial adenomatous polyposis—were defined as familial cases [20]. The presence of a deleterious mutation in a DNA mismatch repair gene at genetic analysis identified HNPCC (or Lynch syndrome); this represented an exclusion criterion. Genetic testing was performed in all EORC patients while LORC patients underwent a genetic test in case of strong family history, presence of genetic syndromes in the family, history of other primary tumors or microsatellite instability (MSI). Due to an internal policy, microsatellite analysis was performed in all cases since 2009.

### 2.3. Statistical Analysis

Categorical and dichotomous variables are presented as number over the total and percentages. The Shapiro–Wilks test was used to test continuous variables for normal distribution (non-normal distribution was considered for *p* < 0.05). Continuous variables are presented as mean ± standard deviation, if normally distributed, or median and interquartile range [IQR], if skewed. Missing data were analyzed for pattern distribution and imputed using a regression-based multiple-imputation model. Pearson’s χ^2^-test with Yeats correction and Fisher’s exact test were used to compare categorical and dichotomous variables. Continuous normally distributed variables were analyzed with a *t*-test, while a Mann–Whitney test was used to compare non-normally distributed variables.

The univariable analysis of the risk factors for incomplete tumoral regression was performed comparing categorical and dichotomous variables a Pearson’s χ^2^-test and continuous variables with an unpaired *t*-test. For each analysis, the odds ratio (OR) or mean difference (MD) with related 95% CI were reported. Multivariable analysis was performed with a back-forward stepwise multivariable binary logistic regression with a significance interval of 0.05. Incomplete tumoral regression was inserted as the dependent variable. An event per variable (EBV) above 10 was considered adequate for limiting the overfitting effect. Variable removal testing was based on the probability of the likelihood ratio statics. The model was tested for fitting using the Omnibus and Hosmer–Lemeshow statistics. For each variable in the model, the OR and 95% CI were reported.

All the analyses were unpaired and two-sided; a *p*-value < 0.05 was considered statistically significant.

Analyses were performed using IBM SPSS Statistics for Windows, Version 24.0 (Armonk, NY, USA: IBM Corp).

## 3. Results

### 3.1. Demographics and Preoperative Data

Out of 1.172 patients treated for RC in the study period, 405 were included in the analysis (326 LORC and 79 EORC) (Figure S1). Gender and smoking habit did not differ.

Mean body mass index was lower in EORC patients (23.77 ± 3.99 vs. 24.87 ± 3.87; *p* = 0.029).

Comorbidities were more reported in LORC patients (70% vs. 22%; *p* < 0.0001). A detailed description of comorbidities is reported in Supplementary Table S1.

Family history for any cancer and for CRC, smoking habit, preoperative and postoperative carcinoembryonic antigen (CEA) did not differ. No differences were found in tumor characteristics before treatment, including distance of the tumor from the anal verge, length of the tumor, intestinal lumen circumferential occupancy, magnetic resonance imaging (MRI)-based tumoral stage, and MRI-based nodal stage.

Most patients in both the cohorts underwent “standard” neoadjuvant CRT. None of the EORC patients underwent SCRT (0% vs. 3%; *p* < 0.0001) but EORC patients received TNT more frequently compared with LORC (15% vs. 1%; *p* < 0.0001). A comparable number and severity of neoadjuvant-related adverse reactions were reported (Table 1). Treatment suspension due to adverse events was reported in 14 LORC cases (4%) while all EORC patients completed the planned treatment.

No differences in the time from the end of neoadjuvant therapy and surgery were found in the two groups.

### 3.2. Response to Neoadjuvant Treatment and Pathological Features

No differences were found between LORC and EORC patients in the pathological tumor classification, status of resection margins, rate of circumferential margin positivity, distance of the tumor from the distal margin, number of harvested lymph nodes, and lymph-node ratio. LORC and EORC patients displayed a similar rate of mucinous and signet-ring cell phenotype. The proportion of patients presenting extramural invasion, lymphovascular invasion, perineural invasion, and tumor deposits was comparable between the cohorts. Although no difference was found in the Dworak regression distribution between LORC and EORC patients, a statistically significant higher proportion of EORC patients showed incomplete regression (Dworak 0–2) compared with LORC patients (49% vs. 35%; *p* = 0.028). A subanalysis on preoperative cT3/T4 cancers undergoing CRT or TNT was also performed (patients undergoing SCRT were excluded from the analysis; no difference in the downstaging from cT3 or cT4 to ypT2/T1/T0 or from cN+ to ypN0 was reported in the two cohorts. The rate of patients undergoing a downstaging bot on T and on N did not differ as well as the Dworak regression distribution. A statistically significant higher proportion of EORC patients showed incomplete regression (Dworak 0–2) compared with LORC (48.6% vs. 35.7%; *p* = 0.044 (Table 2)).

The factors associated with an incomplete regression at univariable analysis included early age of onset (25% vs. 16%; OR = 1.79; 95% CI: 1.09 to 2.94; *p* = 0.028), smoking status (Non-smokers: 46% vs. 55%; Ex-smokers: 21% vs. 23%; Smokers: 32% vs. 21%; *p* = 0.047), pre-neoadjuvant tumoral length (5.44 ± 2.06 vs. 4.83 ± 1.96; MD = −0.60; 95% CI: −1.01 to −0.19; *p* = 0.004), pre-neoadjuvant percentage of lumen occupancy (70 ± 27 vs. 60 ± 27; MD = −9.95; 95% CI: −15.43 to −4.48; *p*< 0.0001), pre-neoadjuvant MRI-based T stage (T2: 6% vs. 13%; T3: 79% vs. 78%; T4: 16% vs. 9%; *p* = 0.016), type of neoadjuvant schedule (Chemoradiotherapy: 88% vs. 96%; Short-course radiotherapy: 4% vs. 1%; Total neoadjuvant: 6% vs. 2%;), extramural invasion (27% vs. 8%; OR = 3.97; 95% CI: 2.24 to 7.04; *p* < 0.0001), lymphovascular invasion (25% vs. 9%; OR = 3.41; 95% CI: 1.93 to 6.03; *p* < 0.0001), perineural invasion (22% vs. 8%; OR = 3.27; 95% CI: 1.80 to 5.93; *p* < 0.0001), and tumor deposits (12% vs. 5%; OR = 2.42; 95% CI: 1.15 to 5.09; *p* = 0.015). The multivariable analysis confirmed early age of onset (OR = 1.83; 95% CI: 1.09 to 3.05; *p* = 0.021), smoking habit (OR = 2.07; 95% CI: 1.26 to 3.39; *p* = 0.004), and extramural invasion (OR = 2.34; 95% CI: 1.18 to 4.66; *p* = 0.004) as independent risk factors for incomplete response to neoadjuvant therapy (Table 3).

## 4. Discussion

This study aimed to compare the pathological response to neoadjuvant therapies between EORC and LORC patients operated for the primary tumor at a single-tertiary center. An incomplete response (Dworak 0–2) was more frequent in EORC patients. Early age of onset resulted to be an independent predictor for a worse response.

Two studies specifically focused on the response to neoadjuvant treatment in EORC [21,22].

Our results are in accordance with the study by Zhang et al. [22] who compared the TRG between a specific subpopulation of locally advanced RC patients aged < 40 years and a cohort of patients aged ≥ 40. The primary endpoint was the rate of pathological complete response (pCR). Authors concluded that young patients with locally advanced rectal cancer had lower pCR rates following neoadjuvant therapies. Young age was also identified as a predictive factor by multivariate analysis. Other predictors for a pCR were tumor size, pre-neoadjuvant cN stage and pre-neoadjuvant CEA levels. In the study by Zhang et al., as in the present study, the TRG was assessed both on the primary tumor and lymph nodes [19]. A retrospective study published by Steinhagen et al. in 2013 [21] analyzing EORC patients failed to identify any differences in the rate of response to neoadjuvant treatment compared with non-age-based cohorts in the literature. However, this work lacked an internal comparative group and presented significant heterogeneity of preoperative workup, neoadjuvant regimens, and interval from treatment end to surgery, severely affecting the generalizability of its findings. Additionally, the classification used to assess the response and if it was evaluated only on the primary tumor or also on lymph nodes was not specified.

In the present study, early age of onset presented as an independent risk factor for an incomplete response to neoadjuvant therapies. Our results may suggest that different characteristics (biological, molecular, and genetic) in this young population can affect the response to current multimodal treatments. The identification of predictive biomarkers for the response to neoadjuvant treatments may help identifying new regimens for a tailored therapy. This aspect is of interest particularly in young patients for which a balance between treatment success, oncologic outcomes and the impact of therapies on quality of life (social, working, and sexual sphere) should be well weighted.

In support of our results, a higher expression of CD133-positive cancer stem cells was found in locally advanced RC patients aged < 40) undergoing neoadjuvant therapies [19]. Current evidence suggests that CD133-positive rectal cancer stem cells are more resistant to chemo–radiotherapy [23,24]. In this study, the authors hypothesized a possible role of CD133-positive cancer stem cells in determining a worse response to neoadjuvant treatments in young RC patients. Furthermore, larger studies are needed to confirm this finding and to explore other factors responsible for the different response to neoadjuvant regimens in young patients.

In our cohort, besides early age of onset, other factors independently associated with an incomplete response included smoking habit and extramural invasion.

Previous studies hypothesized that aggressive pathological features and advanced stage at diagnosis in EORC patients may be the cause for a worse response to neoadjuvant therapies [21,22,25,26,27,28,29]. However, in the present study, and in previous studies by our group [9,10,15], we did not report any difference in pathological features between EO- and LORC patients. Although it is well known that young patients are diagnosed at a more advanced stage [9,10,15], the present study—focused on preoperatively treated locally advanced RC patients—presented a very homogeneous population in terms of pre-treatment MRI stage. Hence, neither pathological features nor stage disease at diagnosis resulted in the peculiar characteristic of EORC patients and could not be hypothesized as causes for a worse response.

Although the preoperative tumor stage did not differ, more EORC patients underwent a TNT regimen, reflecting a tendency to be more “aggressive” in young patients. TNT was initially introduced to improve systemic disease control thanks to a potential early treatment of occult micro-metastases, However, it demonstrated to also improve the rate of complete response [29,30,31,32]. Although more young patients underwent a TNT regimen, a benefit in terms of response to treatment was not observed. The disease-free survival analysis will allow us to determine if TNT regimens improve systemic disease control in EORC patients. SCRT was performed only in 10 (3%) LORC patients as in our institution SCRT is indicated—after multidisciplinary discussion—only to those patients with contraindication to chemotherapy.

The partially retrospective design and the wide time span can be regarded as limitations of this study, as therapeutical approaches have evolved and are still evolving. Another limitation relies on the potential selection bias lead by the disproportion in the number of patients in the two study cohorts. To note, there are several methods to assess the pathological response after neoadjuvant therapies; therefore, results may slightly change according to the TRG system used. This can be regarded as a limitation. However, in this work, we evaluated the TRG both on the primary tumor and the lymph nodes. The classification we used is very similar to the recently proposed modified Dworak classification [33] which assesses both the primary tumor and lymph nodes, and was reported to be a better predictor of survival than other TRG systems for the evaluation of the primary tumor [33]. Therefore, the evaluation of the pathologic response both on the primary tumor and the lymph nodes can also be regarded as a strength of this study. The single-center nature can be both a limitation and a strength because the homogeneity in the therapeutic pathway and the accuracy of the database maintenance allowed for great precision in data collection and retrieval. Additionally, the multidisciplinary decision-making and treatment in a single center allowed a homogeneous evaluation of the disease during all the phases. The strict definition of EORC according to age and the large cohort of sporadic EORC can be regarded as other strengths of the study.

## 5. Conclusions

In conclusion, early age of onset presented as an independent risk factor for incomplete response to neoadjuvant treatment. This result might reflect a different biology of EORC, which in turn affects response to current neoadjuvant therapies, calling for a different multimodal strategy in this group of patients. The identification of biomarkers associated with response or resistance to therapies may help identifying new treatment regimens for a targeted therapy in RC patients. However, the limited literature on the topic, the non-homogeneous endpoints (the rate of pCR or the rate of incomplete response) and the different classification used to assess the pathologic response to neoadjuvant treatment hamper the possibility to draw definitive conclusion on the topic. Further studies with homogeneous endpoints and definitions are needed to eventually confirm our results.

## Figures and Tables

**Table 1:** Baseline and preoperative characteristics, *n* (%), mean ± standard deviations, median (IQR).

Characteristics	LORC	EORC	*p*-Value
Number of patients	326	79	
Age, years	65.23 ± 8.73	43.15 ± 5.04	<0.0001
Gender, females	132 (40%)	37 (47%)	0.312
BMI, Kg/m^2^	24.87 ± 3.87	23.77 ± 3.99	0.029
Smoking status			0.285
Non-smokers	166 (51%)	43 (54%)	
Ex-smokers	71 (22%)	21 (27%)	
Smokers	89 (27%)	15 (19%)	
Preoperative CEA, ng/mL	2.00 [1.10–3.00]	2.00 [1.10–3.00]	0.813
Postoperative CEA, ng/mL	2.16 [1.30–2.16]	2.16 [1.00–2.16]	0.075
Comorbidities	228 (70%)	17 (22%)	<0.0001
Family history of cancer	177 (54%)	38 (48%)	0.379
Family history of CRC	63 (19%)	22 (28%)	0.123
Distance of the tumor from the anal verge, cm	5.00 [4.00–8.00]	5.00 [3.00–6.50]	0.113
Length of the tumor, cm	5.00 [4.00–6.00]	5.00 [4.00–6.00]	0.866
Lumen circumference occupancy, %	62 [40–95]	65 [50–95]	0.453
MRI-based T			0.712
cT2	35 (10.7%)	7 (9%)	
cT3	255 (78%)	61 (77%)	
cT4	36 (11%)	11 (14%)	
MRI-based positive lymph nodes	283 (87%)	69 (87%)	1.000
Type of neoadjuvant therapy			<0.0001
Chemoradiotherapy	312 (96%)	67 (85%)	
Short-course radiotherapy	10 (3%)	--	
Total neoadjuvant therapy	4 (1%)	12 (15%)	
Adverse reactions to neoadjuvant therapy	85 (26%)	24 (30%)	0.480
G1	33 (39%)	10 (42%)	
G2	32 (38%)	12 (50%)	
G3	14 (16%)	2 (8%)	
G4	6 (7%)	--	
Unplanned neoadjuvant interruption/modification	18 (5%)	3 (4%)	0.778
Suspension	14 (77%)	--	0.082
Reduction or modification	4 (23%)	3 (100%)	0.138
Time from neoadjuvant end to surgery, days	83 [69–92]	82 [67–90]	0.358

Abbreviations: IQR, interquartile range; LORC, late-onset rectal cancer; EORC, early-onset rectal cancer; BMI, body mass index; CEA: carcinoembryonic antigen; CRC, colorectal cancer; MRI, magnetic resonance imaging. Categorical and dichotomous variables were analyzed with Pearson’s χ^2^ test with Fisher’s exact test. Continuous variables were tested for normality with the Shapiro–Wilks test and analyzed with an unpaired *t*-test if normally distributed or Mann–Whitney test if skewed.

**Table 2:** Histopathological characteristics and response to therapy, *n* (%), median (IQR).

Characteristics	LORC	EORC	*p*-Value
Number of patients	326	79	
Tumoral pathological stage (AJCC, 8th edition)			0.606
Stage 0	77 (24%)	17 (22%)	
Stage I	83 (25%)	16 (20%)	
Stage II	91 (28%)	23 (29%)	
Stage III	75 (23%)	23 (29%)	
Pathological tumoral classification			0.709
ypT0	83 (25%)	19 (24%)	
ypT1	25 (8%)	6 (8%)	
ypT2	79 (24%)	14 (18%)	
ypT3	125 (39%)	36 (46%)	
ypT4	14 (4%)	4 (4%)	
Pathological node classification			0.640
ypN0	240 (74%)	53 (67%)	
ypN1	53 (16%)	16 (20%)	
ypN2	20 (6%)	7 (9%)	
ypN1c	13 (4%)	3 (4%)	
Resection margins status			0.690
R0	319 (98%)	77 (98%)	
R1	7 (2%)	2 (2%)	
Positive circumferential margin	5 (1%)	1 (1%)	1.000
Distance of the tumor from the distal margin, cm	2.00 [1.17–3.00]	2.00 [1.20–2.40]	0.392
Number of lymph nodes harvested	19 [14–24]	20 [15–26]	0.312
Lymph-node ratio	0.00 [0.00–0.00]	0.00 [0.00–0.07]	0.151
Mucinous component	17 (5%)	8 (10%)	0.126
Signet-ring cell component	3 (1%)	2 (2%)	0.252
Extramural invasion	49 (15%)	13 (16%)	0.730
Lymphovascuar invasion	44 (13%)	16 (20%)	0.157
Perineural invasion	39 (12%)	15 (19%)	0.138
Tumor deposits	24 (7%)	7 (9%)	0.640
Microsatellite instability	2 (1%)	2 (2%)	0.172
Mutations (KRAS/BRAF/NRAS/Pi3KCa)	20 (6%)	9 (11%)	0.140
Tumor regression (Dworak + LN classification)			0.124
Grade 0	5 (1%)	1 (1%)	
Grade 1	32 (10%)	12 (15%)	
Grade 2	78 (24%)	26 (33%)	
Grade 3	134 (41%)	23 (29%)	
Grade 4	77 (24%)	17 (24%)	
Incomplete tumor regression	115 (35%)	39 (49%)	0.028
Downstaging from pre-treatment T3/T4	158/291 (54%)	34/72 (47%)	0.294
Downstaging from cN+ to ypN0 *	213/258 (82.5%)	47/63 (75%)	0.149
Downstaging on T and N *	133/291 (45.7%)	28/72 (38.8%)	0.297
Tumor regression (Dworak + LN classification) *			0.219
Grade 0	5 (1.9%)	1 (1.4%)	
Grade 1	29 (10%)	11 (15.3%)	
Grade 2	70 (25%)	23 (32%)	
Grade 3	121 (40.5%)	21 (26.3%)	
Grade 4	66 (22.6%)	16 (25%)	
Incomplete tumor regression	104 (35.7%)	35 (48.6%)	0.044

Abbreviations: IQR, interquartile range; LORC, late-onset rectal cancer; EORC, early-onset rectal cancer; AJCC, American joint committee on cancer; LN lymph nodes. Categorical and dichotomous variables were analyzed with a Pearson’s χ^2^ test with Fisher’s exact test. Continuous variables were analyzed with a Mann–Whitney test. * In T3/T4 patients, short-course RT was excluded.

**Table 3:** Univariable analysis and multivariable logistic regression analysis of the risk factors of incomplete tumor regression.

	Univariable Analysis			Multivariable Analysis		
	OR/MD	95% CI	*p*-Value	OR	95% CI	*p*-Value
Age of onset (vs. LORC)	1.79	1.09 to 2.94	0.028	1.79	1.02 to 3.16	0.042
Gender (vs. male)	0.83	0.55 to 1.25	0.407			
BMI, Kg/m^2^	−0.02	−0.81 to 0.76	0.952			
Smoking status (vs. non-smokers)			0.047			0.030
Ex-smokers				1.15	0.66 to 2.02	0.615
Smokers				2.03	1.19 to 3.45	0.009
CRC familiarity	1.04	0.64 to 1.71	0.900			
Tumor–anal verge, cm	−0.40	−0.97 to 0.17	0.166			
Length of tumor, cm	−0.60	−1.01 to −0.19	0.004	1.12	0.99 to 1.25	0.059
Circumference occupancy, %	−9.95	−15.43 to −4.48	<0.0001	1.01	0.99 to 1.02	0.077
MRI T stage (vs. T2)			0.016			0.531
T3				1.58	0.69 to 3.59	0.270
T4				1.65	0.59 to 4.63	0.336
MRI positive lymph nodes	1.22	0.66 to 2.25	0.547			
Neoadjuvant type (vs. chemoradio)			0.028			0.186
Short-course radiotherapy				4.04	0.88 to 18.47	0.072
Total neoadjuvant therapy				1.91	0.60 to 6.02	0.270
Neoadjuvant interruption	1.24	0.51 to 3.00	0.650			
Diagnosis-surgery, days	−11.15	−31.89 to 9.58	0.290			
Mucinous component	2.11	0.93 to 4.79	0.089			
Signet-ring cells	2.47	0.41 to 14.97	0.373			
Neoadjuvant end-surgery, days	−1.65	−11.39 to 8.09	0.739			
Extramural invasion	3.97	2.24 to 7.04	<0.0001	2.34	1.18 to 4.66	0.015
Lymphovascular invasion	3.41	1.93 to 6.03	<0.0001	1.98	0.99 to 3.97	0.052
Perineural invasion	3.27	1.80 to 5.93	<0.0001	1.31	0.62 to 2.77	0.484
Tumor deposits	2.42	1.15 to 5.09	0.021	1.40	0.59 to 3.31	0.433
Microsatellite instability	0.54	0.06 to 5.24	1.000			
Mutations	1.83	0.86 to 3.89	0.163			

Abbreviations: OR, odds ratio; MD, mean difference; 95% CI, 95% confidence intervals; LORC, late-onset rectal cancer; BMI, body mass index; CRC, colorectal cancer; MRI, magnetic resonance imaging. Categorical and dichotomous variables were analyzed with a Pearson’s χ2 test with Fisher’s exact test. Continuous variables were analyzed with an unpaired *t*-test. The logistic regression model was statistically significant (omnibus test: χ^2^_(14)_ = 63.06; *p* < 0.0001). The model explained 19% (Nagelkerke R^2^) of the variance of the incomplete regression rate and correctly classified 68% of the cases. The Hosmer–Lemeshow goodness-of-fit test of the final model (χ^2^_(8)_ = 4.63; *p* = 0.796) indicated an adequate fitness.

## Data Availability

The datasets analyzed in the current study are available from the corresponding author on reasonable request.

## Supplementary Materials

The following supporting information can be downloaded at https://www.mdpi.com/article/10.3390/cancers15143750/s1: Figure S1: Study flow chart; Table S1: Patients’ comorbidities.
