# Supplementary material for: Early Age of Onset Is an Independent Predictor for a Worse Response to Neoadjuvant Therapies in Sporadic Rectal Cancer Patients
Source: Cancers (Basel). 2023 Jul 24;15(14):3750. doi: 10.3390/cancers15143750 (PMC10378654; doi:10.3390/cancers15143750)
Supplement: Supplementary file 1 [file cancers-15-03750-s001.zip › cancers-2452348-supplementary.pdf]

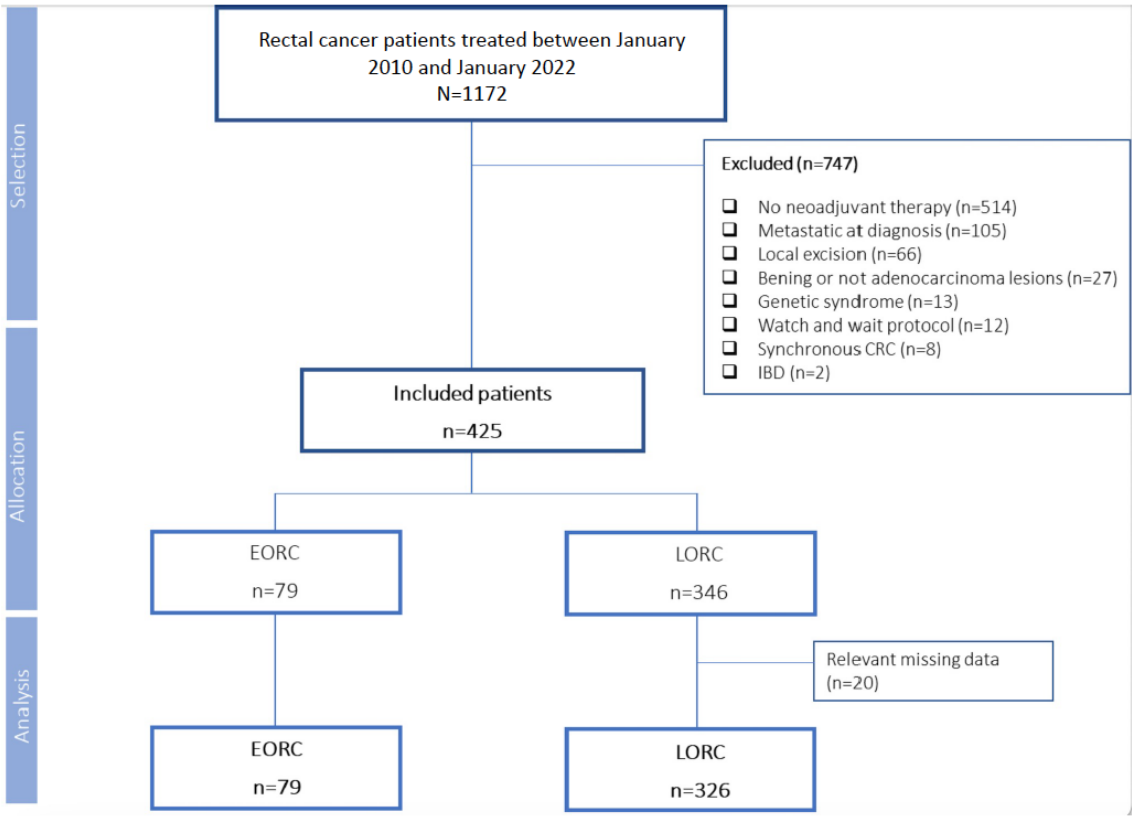

Supplementary Figure S1: Study flow chart

Supplementary Table S1: Patients' comorbidities

Comorbidities	LORC	EORC	*p*-value
Number of patients	228 (47.3%)	17 (22%)	<0.0001
Cardiovascular	108	2	
Pulmonary	8	2	
Metabolic	12	6	
Autoimmune/Rheumatic	24	1	
Cardiovascular & Metabolic	66	0	
Kidney	5	0	
Others	5	6	
